# Local adaptation and phenotypic plasticity in two forest understorey herbs in response to forest management intensity

**DOI:** 10.1093/aobpla/plae061

**Published:** 2024-11-21

**Authors:** Charlotte Møller, Martí March-Salas, Pieter De Frenne, J F Scheepens

**Affiliations:** Plant Evolutionary Ecology, Faculty of Biological Sciences, Goethe University Frankfurt, Max-von-Laue-Str. 13, 60438 Frankfurt am Main, Germany; Botany Unit, Finnish Museum of Natural History, University of Helsinki, Kaisaniemenranta 2 FI-00014, Finland; Plant Evolutionary Ecology, Faculty of Biological Sciences, Goethe University Frankfurt, Max-von-Laue-Str. 13, 60438 Frankfurt am Main, Germany; Area of Biodiversity and Conservation, Department of Biology and Geology, Physics and Inorganic Chemistry, University Rey Juan Carlos-ESCET, Tulippán s/n. 28933 Móstoles, Madrid, Spain; Instituto de Investigación en Cambio Global (IICG-URJC), Universidad Rey Juan Carlos, Calle Tullipán s/n 28933, Móstoles, Madrid, Spain; Forest & Nature Lab, Faculty of Bioscience Engineering, Ghent University, Geraardsbergsesteenweg 267, 9090 Gontrode, Belgium; Plant Evolutionary Ecology, Faculty of Biological Sciences, Goethe University Frankfurt, Max-von-Laue-Str. 13, 60438 Frankfurt am Main, Germany

**Keywords:** *Anemone nemorosa*, local adaptation, *Milium effusum*, phenotypic plasticity, reciprocal transplant

## Abstract

Local adaptation is a common phenomenon that helps plant populations to adjust to broad-scale environmental heterogeneity. Given the strong effect of forest management on the understorey microenvironment and often long-term effects of forest management actions, it seems likely that understorey herbs may have locally adapted to the practiced management regime and induced environmental variation. We investigated the response of *Anemone nemorosa* and *Milium effusum* to forest management using a transplant experiment along a silvicultural management intensity gradient. Genets were sampled from sites with contrasting management intensities and transplanted sympatrically, near allopatrically and far allopatrically along the management intensity gradient to test for local adaptation and phenotypic plasticity, as well as to sites where the species were absent to test for recruitment versus dispersal limitations. We then measured survival and fitness traits over two growing seasons. We found only little evidence of local adaptation in *A. nemorosa* and *M. effusum*, whereas various traits in both species showed linear plastic changes in response to transplantation along the forest management intensity gradient. Furthermore, *A. nemorosa* performed worse when transplanted to unoccupied sites, suggesting recruitment limitation, whereas *M. effusum* performed better in unoccupied sites, suggesting dispersal limitation. Altogether, our results underpin the importance of forest management to indirectly drive phenotypic variation among populations of forest plants.

## Teaser

This study explores how forest management, and the underlying environmental variables impacts local adaptation and phenotypic plasticity in two understorey herbs, Anemone nemorosa and Milium effusum. Using a transplant experiment along a gradient of silvicultural management intensity, the plants’ survival and fitness in familiar and novel environments was assessed. Results showed minimal local adaptation but substantial phenotypic plasticity in both species, indicating that forest management practices influence their trait variation. Additionally, while A. nemorosa struggled to establish in unoccupied areas, suggesting recruitment limitation, while M. effusum seemed to be more limited by dispersal.

## Introduction

Adaptation to local environmental conditions plays a fundamental role in the maintenance of genetic diversity (Blanquart *et al.* 2013), a type of diversity that is being jeopardized due to land-use transformations (Exposito-Alonso 2023). Whereas adaptation of plant populations to their local environment has been demonstrated in numerous studies (Leimu and Fischer 2008; Hereford 2009; Pluess *et al.* 2011; Ågren and Schemske 2012; Bennington *et al.* 2012; De Frenne *et al.* 2012, 2014b; Meineri *et al.* 2013; Frei *et al.* 2014), to our knowledge, local adaptation of understorey herbs to forest management remains unexplored to date, despite the common occurrence of forest habitat worldwide and strong management-dependent environmental variation within many forests (Willems *et al*. 2021; Møller *et al.* 2023a).

Forest management actions affect forest structural attributes directly by altering tree species composition, crown projection area and structural complexity, which, in turn, has strong effects on the biotic and abiotic forest understorey environment, including the microclimate (Willems *et al.* 2021; Møller *et al.* 2023a). For example, structural complexity is an index based on the horizontal and vertical profiles of the forest (Ehbrecht *et al.* 2017), with structurally more complex forests often being more heterogeneous and providing support to ecosystem stability, resilience and biodiversity (Campbell *et al.* 2009; Dolezal *et al.* 2020). In addition, soil properties are also under strong influence by forest management regimes. Canopy openings caused by tree felling can lead to nutrient depletion of the soil (De Frenne *et al.* 2013), and the microclimate strongly affects soil decomposition (Brabcová *et al.* 2022). Furthermore, below-ground environmental factors such as soil pH can be affected by the dominant tree species, which is often determined by management (Augusto *et al.* 2004; Møller *et al.* 2023a). The understorey environment can, therefore, be described as an environment with strong management-imposed constraints.

Understorey herbs play a key role in temperate forest ecosystems (Gilliam 2007; Landuyt *et al.* 2019), as they harbour the highest plant biodiversity of all the forest strata and provide important ecosystem services (Gilliam 2007). Understorey herbs are crucial for nutrient cycling of the forest, and they also contribute to the net primary productivity and annual litter fall (Gilliam 2007). Previous studies have shown that understorey herbs can be affected greatly by forest structural attributes, not only in species composition and diversity (Valdés *et al.* 2015) but also in phenology (Willems *et al.* 2021), and in the genetic basis of ecologically important phenotypic traits (Møller *et al.* 2023a). Due to the strong effect of management on the forest understorey environment, we can furthermore expect that part of the observed genetically based phenotypic variation in forest understorey herbs (Møller *et al.* 2023a) is due to local adaptation to forest management and its effects on the understorey microenvironment.

Local adaptation is a result of divergent selection and evolution, equipping individuals with a fitness advantage under their local environmental conditions compared to foreign environments (Kawecki and Ebert 2004). Thus, resident genotypes should perform better in their local habitat than genotypes originating from other habitats (Kawecki and Ebert 2004). The ‘gold standard’ to study local adaptation is through reciprocal transplant experiments (Gibson *et al.* 2016; Johnson *et al.* 2022), as it allows to separate environmental effects from source of origin effects and to investigate interactions between them (i.e. Genotype × Environment). One way to study local adaptation is by sympatric–allopatric comparisons (where sympatric means individuals for which origin and transplant site match, and allopatric means individuals for which their origin and transplant site do not match), allowing the strength of local adaptation to be investigated while taking both habitat and population differences into account (Blanquart *et al.* 2013). As an extension to simple site and population comparisons, environmental differences between sites can be considered, for instance, by separating the allopatric component into different degrees of dissimilarity to sympatric combinations (Adiba *et al.* 2010), for example, sympatric versus near allopatric (small environmental difference from sympatric transplant) and sympatric versus far allopatric (large environmental difference from sympatric transplant; Kesselring *et al.* 2019). The implementation of such degrees of allopatric transplantations (near and far transplantation) also opens up possibilities to investigate the effects of particular environmental factors, even along continuous scales (Gorton *et al*. 2022).

Forest understorey herbs are generally dispersal limited, and species are often absent in forest patches due to either dispersal or recruitment limitations (Hermy and Verheyen 2007). Previous studies have confirmed that phenotypic variation and their genetic and plastic components play important roles in the adaptation of plants to future climatic conditions (Arndt *et al*. 2001; Lafitte *et al.* 2007; Møller *et al.* 2023b). The transplantation of individuals into dissimilar environments in combination with investigations of phenotypic variation and plant performance after transplantation could provide useful insights in dispersal and recruitment limitations (Spiecker 2003). Furthermore, by adding environmental data, the degree of dissimilarity of a foreign habitat to focal species can be estimated, and transplantation experiments can be used to understand that factors limit the colonization of sites, which can be valuable information for ecological restoration (Ehrlén and Eriksson 2000).

Here, we apply a reciprocal transplant experiment to test whether populations of two common European forest understorey herbs—*Anemone nemorosa* and *Milium effusum*—are locally adapted to forest management and to local environmental variables. In addition, we experimentally test if sites are unoccupied because of recruitment limitations or due to limited dispersal abilities of the species. We expect to observe species differences in local adaptation in line with their dispersal abilities and gene flow. More specifically, stronger local adaptation is expected in species with comparatively low dispersal ability and reduced gene flow—such as *Anemone nemorosa*—compared to species with comparatively high dispersal ability and strong gene flow—such as *Milium effusum* (see “Materials and Methods” section). Furthermore, we expect dispersal limitation in *A. nemorosa*, the species with relatively low dispersal abilities, whereas we expect recruitment limitations in *M. effusum*, the relatively well-dispersing species.

## Materials and Methods

### Study species

We used two perennial plant species of the understorey herb community, *Anemone nemorosa* (Ranunculaceae) and *Milium effusum* (Poaceae). The two study species have a large distribution range, occurring commonly in temperate forests throughout Europe and Western Asia (De Frenne *et al.* 2010, 2017).

*Anemone nemorosa* is an early flowering forb, relying heavily on clonal spread through rhizomes (Frederiksen and Rasmussen 2006). It is shade tolerant, growing primarily in temperate woodland conditions where its yearly reproductive cycle is suited to the leaf-out dynamics of the forest (Shirreffs 1985). Despite the species having a low colonization rate of <1 m year^−1^ (Brunet *et al.* 2012), sexual reproduction is still considered important for population persistence (Frederiksen and Rasmussen 2006). However, genetic differentiation among 20 populations in Switzerland was found to be substantial (*G*_ST_ = 0.29), likely reflecting low historical gene flow among populations (Stehlik and Holderegger 2001).

*Milium effusum* is a tall-growing, shade-tolerant tussock grass that typically grows in temperate deciduous forests, but it can also occur along railways and roads (De Frenne *et al.* 2017). It flowers in summer and relies on wind for dispersal of its seeds, but re-sprouting through short stolons also supports colonization (De Frenne *et al.* 2011). This vegetative spread combined with seed dispersal results in a high overall colonization rate of 2.78 m year^−1^ (Brunet *et al*. 2012). Nevertheless, a study by Tyler (2002) revealed substantial genetic differentiation amongst 21 southern Swedish and 23 northern Swedish populations (*G*_ST_ = 0.29 and *G*_ST_ = 0.53, respectively).

### Experimental design

We conducted a reciprocal transplant experiment on a total of 39 sites in all 3 regions of the research platform Biodiversity Exploratories (www.biodiversity-exploratories.de) distributed across Germany: Schwäbische-Alb (ALB), Hainich-Dün (HAI) and Schorfheide-Chorin (SCH) (Fischer *et al.* 2010; **see Supporting Information—Table S1**). Each region contains 50 forest sites of 100 × 100 m, ranging from protected and natural areas to highly managed timber plantations and we consider the regions as replicates. All forest sites have consistently been managed at individual management intensities and have been monitored by the Biodiversity Exploratories for at least 15 years. Although we do not know the history of the conducted management regimes beyond the last 15 years, the constancy in management per site during the 15 years suggests continuity in the further past.

Schwäbische-Alb is characterized by calcareous bedrock and mostly contains small-scale mosaic forests, containing both coniferous and deciduous tree species. Schwäbische-Alb has the highest elevation of the three regions, up to 860 m, and despite being the most southern region, it has the coldest annual mean temperature of 6–7 °C and a mean annual precipitation of 700–1000 mm (Fischer *et al.* 2010). The mountain ranges are rich in limestone, and in combination with steep reliefs, erosion leads to shallow soils with low water storage potential. Schwäbische-Alb is mostly characterized by small-scale mosaic forests and high management intensity. Hainich-Dün is located in the centre of Germany and is also characterized by calcareous bedrock. Hainich-Dün has a maximum elevation of 550 m, a mean annual temperature of 6.5–8 °C and a mean annual precipitation of 500–800 mm. Hainich-Dün is one of the largest connected deciduous forest areas in Germany, mainly characterized by extensive beech forests, and is dominated by selection forest management (Fischer *et al.* 2010). Schorfheide-Chorin is located in the northern part of Germany and is a young glacial landscape with a maximum elevation of 140 m. With an annual mean temperature of 8–8.5 °C, it is the warmest of the three regions, and with a mean annual precipitation of 500–600 mm, it is also one of the driest regions in Germany (Fischer *et al.* 2010). Schorfheide-Chorin is characterized by sandy areas and the majority of the forests are age-class, consisting of old natural beech forests, mixed forests, but also intensively managed pine monocultures (Fischer *et al.* 2010).

**Figure 1.**
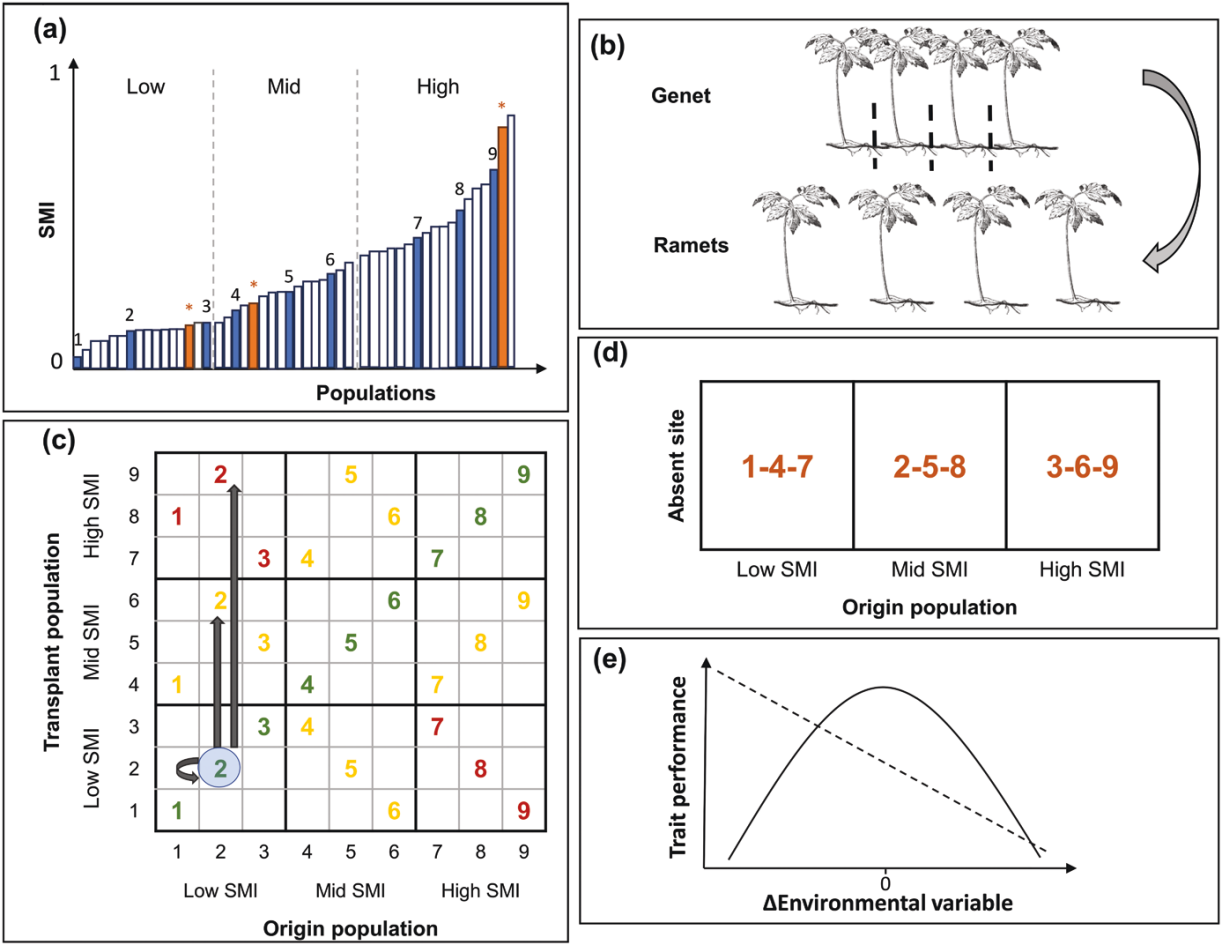
Schematic of the experimental design representing an example of a single region and species. (A) For each region, all 50 sites were ranked from low to high forest management intensity (SMI). We then created equally broad bins (in terms of SMI distance) for low, mid and high SMI populations, and we chose a total of nine populations of origin, three from each bin (restricted to sites we knew the focal species occurred), replicated three times (numbered bars). (B) We sampled six genets from each population and propagated each genet into four ramets. (C) Sympatric–allopatric comparisons were conducted for each replicate population (1–9) by transplanting ramets of all genets sympatrically (green), near allopatrically (yellow) and far allopatrically (red). (D) Sympatric—absent comparisons were conducted for each replicate population by choosing a site (orange bars labeled with stars in panel a) from each bin (low, mid and high) where the species did not naturally occur and to which the fourth ramet of each replicate was transplanted (population 1,4 and 7 to a low SMI absent site; population 2,5 and 8 to a mid-SMI absent site, and population 3,6 and 9 to a high SMI absent site). (E) Possible results from the reciprocal transplant experiment visualized on a continuous environmental scale. A concave relationship (solid line) with trait performance highest at 0 difference of the environmental variable (Δ) between sympatric and allopatric sites would indicate local adaptation whereas a linear relationship (dashed line) would reflect linear phenotypic plastic responses to environmental gradients (Gorton *et al.* 2022).

In order to capture management and disturbance levels caused by forest management, Schall and Ammer (2013) developed an index for forest management intensity, called SMI (silvicultural management intensity). Silvicultural management intensity is calculated using a function incorporating a stand density component and a risk of stand loss component, which, in turn, are derived from the tree species, stand age and aboveground living and dead wooden biomass. The risk of stand loss component encompasses the combined effect of tree species selection and stand age, whereas the stand density component accounts for the effects of removals and regeneration methods (Schall and Ammer 2013). Silvicultural management intensity ranges between 0 and 1, with 0 indicating no management and 1 intensive management. Typically, monoculture forests rank from the lowest to the highest management intensity based on their traditional rotation period and the cultivated species as follows: oak < beech < pine < spruce (Schall and Ammer 2013). Silvicultural management intensity values were calculated based on the last 15 years of forest management at the 50 forest sites per region. To cover the whole gradient of forest management intensity (SMI) within each region, we chose our sites of origin by ranking all 50 sites from each region from low to high management intensity (Fig. 1A). Within regions, SMI was variable among the sites and did not show any spatial gradients. We then divided the SMI gradient of all sites in each region into low, mid and high management intensity bins and, per species, selected three sites from each bin in which the species was present. In each of the three regions, we thus selected nine populations of each species (three populations per SMI bin). Where possible, we selected sites in which both species co-occurred. We also selected one site per SMI bin where the focal species did not naturally occur. In spring 2020, we collected six genets within each selected population as replicates of that population. To avoid sampling genetically identical plants, each individual was sampled with a minimum distance of 10 m to the next sampled plant (Holderegger *et al.* 1998). Each genet was split into four separate ramets (Fig. 1B) and potted into multitrays (5 cm ø and 5.5 cm deep, 54 pots per tray) filled with potting soil (CL T torffrei, Einheitserde, Sinntal-Altengronau, Germany) and placed outdoors. A ramet of *A. nemorosa* consisted of a piece of rhizome ca. 4 cm long buried in the soil whereas a cutting of *M. effusum* consisted of a single shoot with roots.

In October 2020, the ramets were transplanted back into sites within their own regions to test for local adaptation: sympatrically into their population of origin and allopatrically into a near and a far allopatric site (Fig. 1C), as well as to a site where the species did not naturally occur (absent) to test for dispersal versus recruitment limitations (Fig. 1D). On each site, the ramets were transplanted within a fenced area to prevent damage by large animals. Each ramet was taken out of the multitray, planted into a 1.5 L pot with drainage holes in the bottom and filled with local soil from the site, and the pot was dug into the soil such that the top of the pot was at ground level [**see Supporting Information Information—****Fig. S1**]. Growing the individuals in pots beneath the soil prevented possible intermingling of rhizomes, while allowing for interactions with microbiota and natural ground water dynamics. In total, we transplanted 648 *A. nemorosa* individuals (3 regions × 9 origins × 6 genets × 4 ramets). However, due to low abundance of *M. effusum* in Schorfheide-Chorin, we could only sample and reciprocally transplant this species in the two other regions, adding up to 432 *M. effusum* individuals (2 regions × 9 origins × 6 genets × 4 ramets).

### Measured traits

Plant measurements were taken in 2021 and 2022. Each year, measurements for *A. nemorosa* were taken during the flowering period of this species in April over the course of 1 week, and measurements for *M. effusum* in the flowering period in June over the course of 1 week. We recorded mortality, which was assumed if no ramets were visible in the pot and was scored as 0, whereas if ramets were visible we scored this as 1. The originally transplanted ramets produced new ramets during the experiment, and we considered each vegetative or flowering shoot of *A. nemorosa* as well as each shoot of *M. effusum* as a single ramet. For each pot, we measured plant height to the nearest 0.5 cm on the tallest ramet and counted the total number of ramets. Furthermore, we counted the number of flowers as the sum of flower buds, flowers and fruits, which we used as a measure of sexual reproductive success. Furthermore, in 2022, we collected the total aboveground biomass for each pot and dried it in a drying oven at 65 °C for 48 h before we weighed it. Correlations among the studied plant traits are available in **Supplementary Information—Fig. S2**. Given that both *A. nemorosa* and *M. effusum* reproduce vegetatively, albeit to different degrees, the number of ramets is an important measurement of vegetative spread and therefore of fitness, and likewise is the height of ramets, biomass, and—as a measure of sexual reproductive fitness—the number of flowers (Younginger *et al.* 2017).

### Environmental variables

As explanatory environmental variables, we considered SMI, structural complexity of the forest stand (an index quantifying stand structural complexity based on the fractal dimension of cross-sectional polygons; Ehbrecht *et al*. 2017), soil pH and the mean annual spring temperature as these environmental variables are known to be strongly affected by forest management and forest structural attributes [**see Supporting Information—****Fig. S3**] and have previously been found to explain the majority of phenotypic variation in the field (Willems *et al.* 2021) and in a common garden experiment (Møller *et al.* 2023a) for the same species. Silvicultural management intensity was included to investigate potentially indirect effects of silvicultural management on the transplanted plants, whereas the other variables were included to investigate potentially direct effects on the transplanted plants, where these variables may themselves be affected by SMI (Willems *et al.* 2021; Møller *et al.* 2023a). All environmental variables were extracted for each site for both 2021 and 2022 from the BExIS2 (www.bexis.uni-jena.de) database, maintained by the Biodiversity Exploratories (Fischer *et al.* 2010). Next, we calculated environmental difference (Δ) for each environmental variable as the difference from each plant’s site of origin to that plant’s site of transplantation. Transplantation to the home site would thus result in zero environmental difference. Furthermore, temperature at +15 cm, 0 cm, and −8 cm relative to the soil surface as well as soil moisture content were measured every 15 min for the whole duration of the experiment using one TOMST TMS4 logger per site (Wild *et al.* 2019).

### Data analyses

All statistical analyses were conducted with R (version 4.1.2; R Core Team 2021). Our dataset was subset for two subsequent analyses. One dataset contained the sympatric–allopatric transplants (to test for local adaptation), while the other contained the sympatric-absent transplants (to test for recruitment vs. dispersal limitation) [see Supporting Information—R Code].

First, we tested for differences in all traits among the categories of sympatric and near and far allopatric transplants. Due to near allopatric sites and far allopatric sites not showing any differences in plant responses, we decided to pool the near and far allopatric sites together and hereafter call them ‘allopatric’ [see Supporting Information—Table S2]. We thus ran five generalized linear mixed-effects models (GLMMs), one for each trait for the sympatric versus allopatric comparisons, using the function ‘lmer’ from the package ‘lme4’ (Bates *et al.* 2007). The measured traits were used as response variables, where a binomial family was applied for survival (i.e. the opposite of mortality), Poisson family was applied for the number of flowers, and Gaussian family for plant height, number of ramets and biomass. The transplant category (sympatric and allopatric) was used as the explanatory variable. Region, population and genet were set as nested random factors. Similarly, we tested for differences between sympatric transplants and plants transplanted to sites where the species was absent. To do so, we ran five GLMMs, one for each trait, for the sympatric versus absent comparisons, using the function ‘glmer’ from the package ‘lme4’ (Bates *et al*. 2007). The measured traits were used as response variables, and the transplant category (sympatric and absent) was used as the explanatory variable. Region, population and genet were set as nested random factors.

Second, to test for local adaptation (sympatric–allopatric comparison) and for recruitment versus dispersal limitations (sympatric-absent comparison), we tested for the effects of environmental differences (Δ) among the origin and transplant sites. We ran five GLMMs, one for each trait, using the function ‘glmer’. The measured traits were used as response variables, where a binomial family was applied for survival, Poisson family was applied for the number of flowers, and Gaussian family for plant height, number of ramets and biomass. The linear and quadratic terms of ΔSMI, Δstructural complexity, Δsoil pH and Δyearly mean spring temperature between origin and transplant sites were included as the explanatory variables. A concave quadratic relationship with its peak trait value around zero environmental differences would indicate local adaptation (Fig. 1E; Gorton *et al*. 2022), because plants would grow increasingly worse in environments that deviate from their home environment. Alternatively, wherever a linear relationship (Fig. 1E) appears, this can be interpreted as a linear phenotypic plastic response along an environmental gradient, for instance reflecting that fitness increases in more benign environments. Genet, population and region were included as nested random factors. Variance inflation factors of the environmental variables were checked and all ranked below 1.5, indicating no to low correlation among the variables (James *et al.* 2013). Due to the testing of multiple models, a Bonferroni correction was applied, lowering the significant *P* value threshold of 0.05 to 0.00125 (Miller 1966). Furthermore, due to the scarcity of significant results in 2021 compared to 2022 [see Supporting Information—Table S3], possibly due to plants not having accumulated significant differences in their performance by 2021, we only investigated the effects of the environmental variables for 2022.

All model test results were obtained by applying the function ‘Anova’ from the package ‘car’ (Fox *et al.* 2012). Shapiro–Wilk and Bartlett tests were used to assess whether assumptions of normality and homoscedasticity of the model residuals were met, respectively. When the assumption of normality of residuals was violated, the response variable was transformed with either logit or the square root to improve the distribution of residuals.

## Results

### Mortality

**Figure 2.**
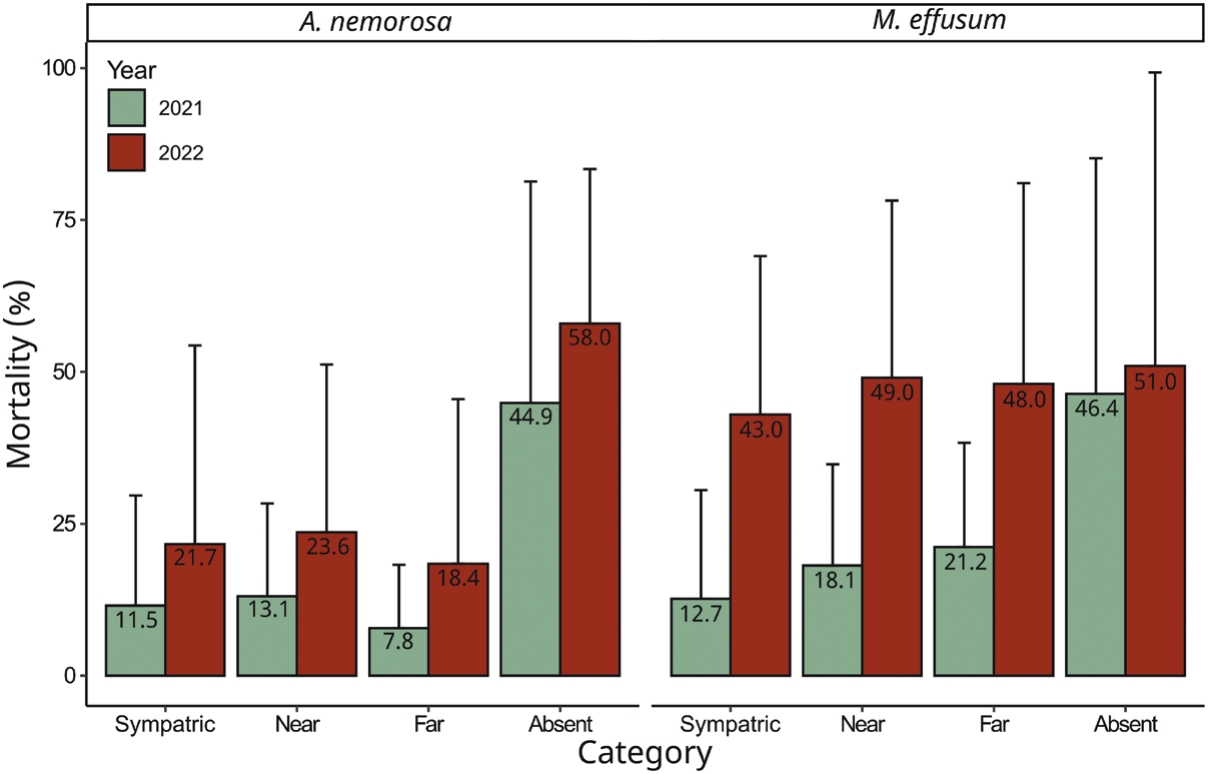
The percentage mortality for the transplant categories sympatric, near allopatric, far allopatric and absent for both years (compared to the start of the experiment) for the study species *Anemone nemorosa* and *Milium effusum*. Numbers within the bars indicate the means across populations. The error bars indicate the standard error across populations.

Mortality was documented first in 2021, when populations had 45–46 % losses in both species in the absent sites, while much lower percentages of mortality were observed in the sympatric and allopatric sites (8–21 %; Fig. 2). In 2022, additional mortality at sympatric, near allopatric and far allopatric sites was lower for *A. nemorosa* (10–11 % of the initial transplants; a 2.0-fold increase of mortality in 2021) compared to *M. effusum* (27–31 %; a 2.7-fold increase of mortality in 2021; Fig. 2). Furthermore, additional mortality in the absent sites was 13 % for *A. nemorosa* and 5 % for *M. effusum* (Fig. 2).

### Sympatric–allopatric comparisons

**Figure 3.**
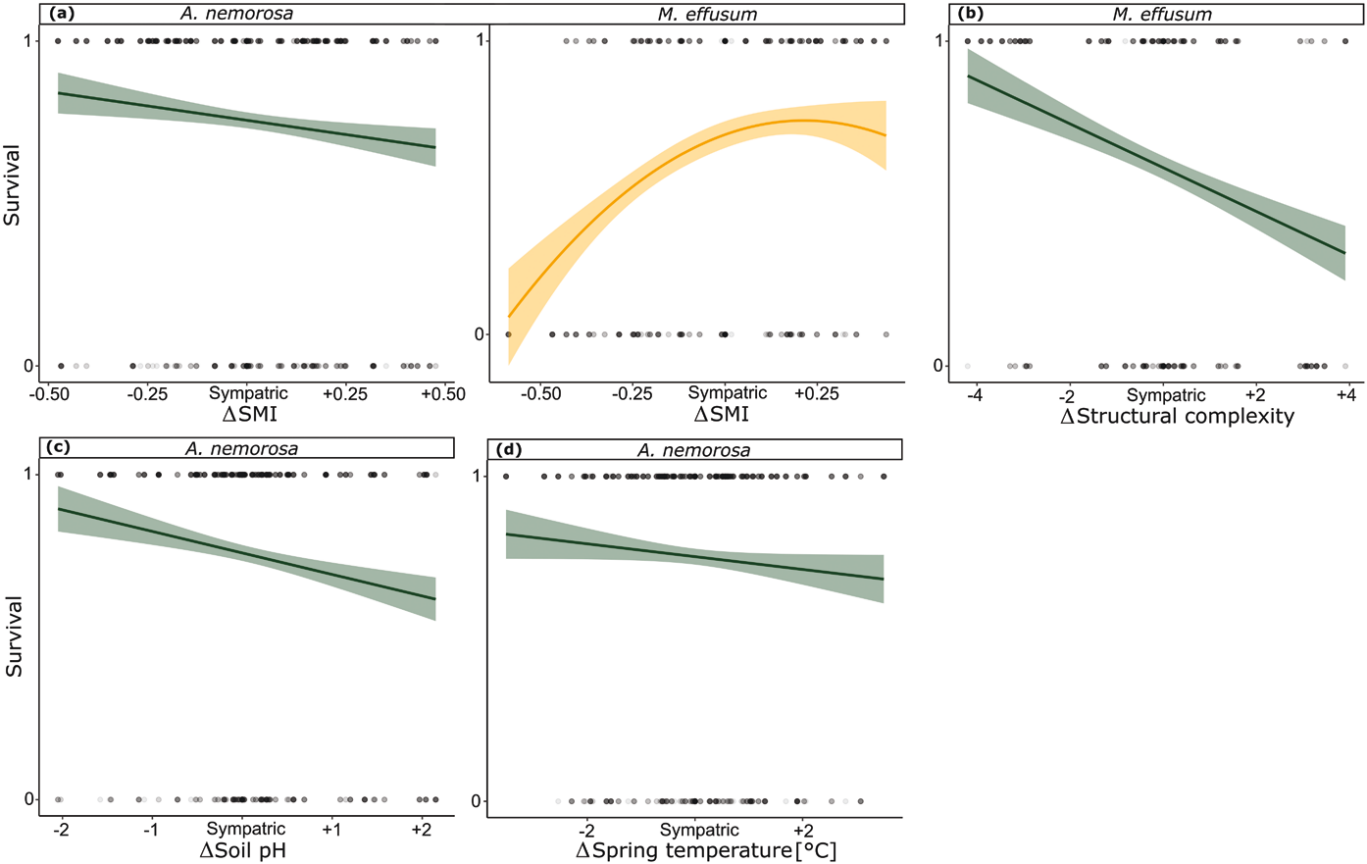
Significant relationships between survival and the environmental differences of sympatric–allopatric comparisons in 2022. Δ Denotes the environmental differences between sites of origin and transplant sites for (A) forest management intensity (SMI) for both *Anemone nemorosa* and *Milium effusum*, (B) structural complexity for *M. effusum* (C) soil pH for *A. nemorosa* and (D) spring temperature for *M. effusum*. Linear regression fits are straight (a, b, c, d) and quadratic fits are curved (a). 95% Confidence intervals are shown around the curves. The data points have been scaled for each trait and species separately.

**Figure 4.**
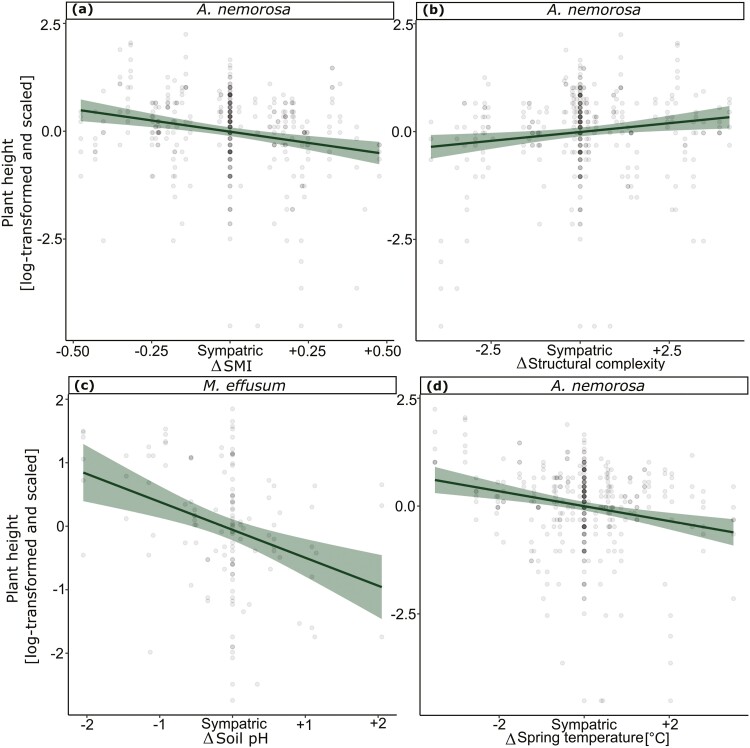
Significant relationships between plant height and the environmental differences of sympatric–allopatric comparisons in 2022. Δ denotes the environmental differences between sites of origin and transplant sites for (A) forest management intensity (SMI) for *Anemone nemorosa*, (B) structural complexity for *A. nemorosa*, (C) soil pH for *Milium effusum* and (D) spring temperature for *A. nemorosa.* Linear regression fits are straight (a, b, c, d) and quadratic fits are curved. 95% Confidence intervals are shown around the curves. The data points have been scaled for each trait and species separately. The *y* axis has been log-transformed and scaled to mean = 0 and SD = 1.

**Figure 5.**
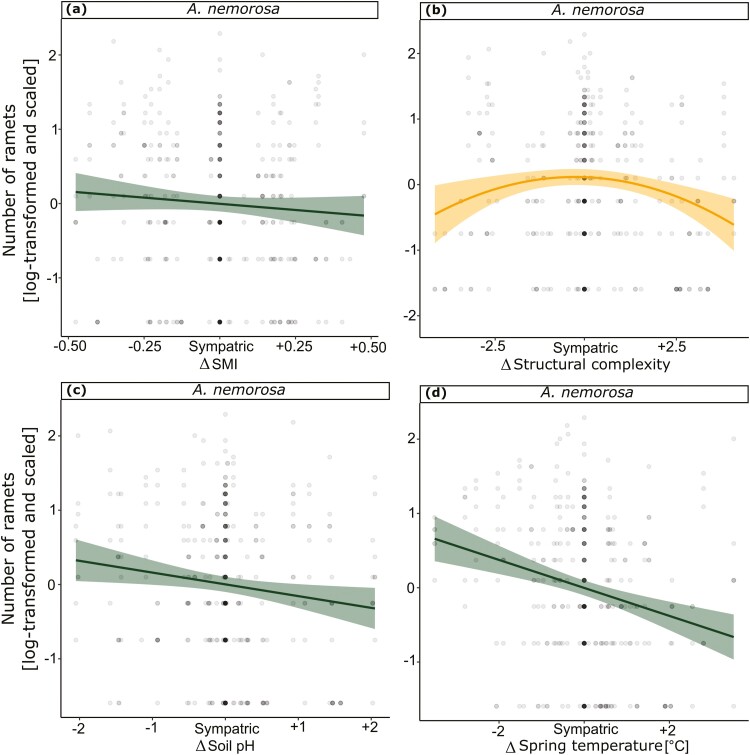
Significant relationships between number of ramets and the environmental differences of sympatric–allopatric comparisons in 2022. Δ denotes the environmental differences between sites of origin and transplant sites for (A) forest management intensity (SMI) for *Anemone nemorosa*, (B) structural complexity for *A. nemorosa*, (C) soil pH for *A. nemorosa* and (D) spring temperature for *A. nemorosa*. Linear regression fits are straight (a, c, d) and quadratic fits are curved (b). 95% confidence intervals are shown around the curves. The data points have been scaled for each trait and species separately. The *y* axis has been log-transformed and scaled to mean = 0 and SD = 1.

**Figure 6.**
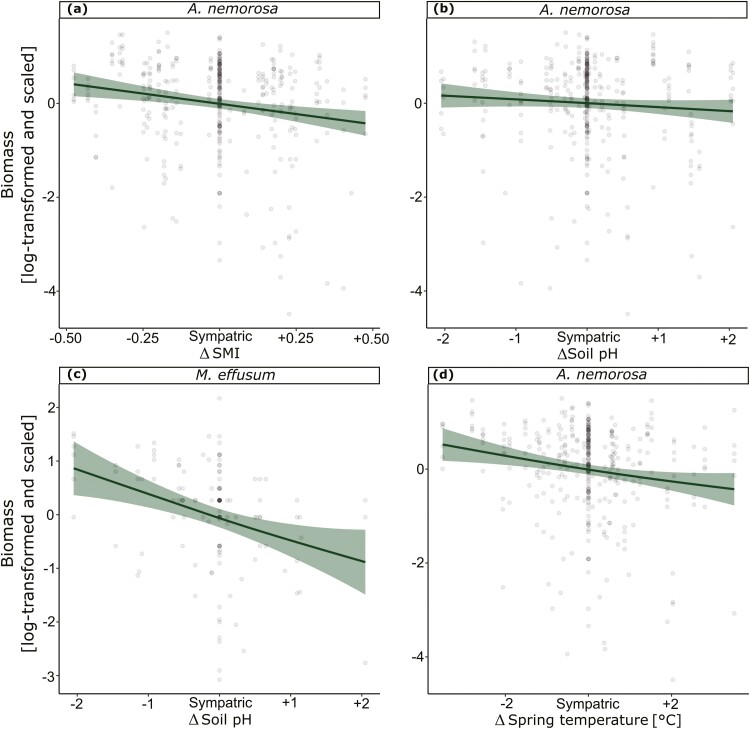
Significant relationships between biomass and the environmental differences of sympatric–allopatric comparisons in 2022. Δ denotes the environmental differences between sites of origin and transplant sites for (A) forest management intensity (SMI) for *Anemone nemorosa*, (B) soil pH for *A. nemorosa*, (C) soil pH for *M. effusum* and (D) spring temperature for *A. nemorosa*. Linear regression fits are straight (a, b, c, d) and quadratic fits are curved. 95% Confidence intervals are shown around the curves. The data points have been scaled for each trait and species separately. The *y* axis has been log-transformed and scaled to mean = 0 and SD = 1.

Tests for population differences between the sympatric and allopatric transplant sites revealed no significant differences in the measured traits in 2021 except for survival in *A. nemorosa* in 2021 and in both species in 2022 (Table 1), but effect sizes are small in comparison to sympatric-absent comparisons (Fig. 2). When testing for effects of environmental differences (Δ) between population origins and transplant sites on the five measured traits in 2022, we found a significant negative linear effect for survival in *A. nemorosa* with ΔSMI (Fig. 3A), Δsoil pH (Fig. 3B) and Δspring temperature (Fig. 3C). Meanwhile *M. effusum* showed a negative linear relationship for survival with ΔStructural complexity (Fig. 3D) and a significant concave relationship with ΔSMI (Fig. 3A), indicating local adaptation. Plant height showed a significant negative linear effect of ΔSMI (Table 2, Fig. 4A) and of Δspring temperature (Fig. 4D) for *A. nemorosa* and of Δsoil pH for *M. effusum* (Fig. 4C). A positive linear effect of Δstructural complexity was found for the plant height of *A. nemorosa* (Fig. 4B). The number of ramets showed a significant quadratic relationship with Δstructural complexity for *A. nemorosa* (Table 3), which was concave and thus indicating local adaptation (Fig. 5A). Furthermore, significant negative linear effects of ΔSMI (Fig. 5B), Δsoil pH (Fig. 5C) and Δspring temperature (Fig. 5D) were found for the number of ramets of *A. nemorosa*, indicating phenotypic plastic responses along these environmental gradients. Finally, biomass of *A. nemorosa* showed significant negative linear effects of ΔSMI (Fig. 6A), Δsoil pH (Fig. 6B) and Δspring temperature (Fig. 6D). Biomass of *M. effusum* also showed significant negative linear effects of Δsoil pH (Fig. 6C). No significant effects were found for number of flowers in either of the study species.

**Table 1.** Results of mixed-effects models testing for trait differences between sympatric–allopatric and sympatric-absent categories in *Anemone nemorosa* and *Milium effusum* in 2021 and 2022. The five measured traits were used as a response variable and the transplant category (i.e. sympatric–allopatric, or sympatric-absent) as the explanatory variable. Region, population and genet were set as nested random factors (residual variances range from 0.034 to 9.372). Marginal *R*^2^ (*R*_*m*_) and conditional *R*^2^ values for the models are given. Significant *P* values after applied Bonferroni-correction are indicated in bold.

	*Anemone nemorosa*								*Milium effusum*							
	2021				2022				2021				2022			
	*χ* ^2^	*P* value	*R*_*m*_	*R*_*c*_	*χ* ^2^	*P* value	*R*_*m*_	*R*_*c*_	*χ* ^2^	*P* value	*R*_*m*_	*R*_*c*_	*χ* ^2^	*P* value	*R*_*m*_	*R*_*c*_
Response variable			Sympatric–Allopatric													
Survival	43.54	**<0.001**	0.042	0.216	125.22	**<0.001**	0.090	0.157	5.22	0.074	0.011	0.164	27.15	**<0.001**	0.040	0.114
Plant height	2.16	0.339	0.003	0.476	2.45	0.294	0.005	0.195	1.88	0.390	0.009	0.009	0.73	0.695	0.005	0.193
Number of ramets	0.71	0.703	0.002	0.110	1.65	0.439	0.004	0.086	0.98	0.613	0.004	0.188	0.04	0.980	0.000	0.168
Number of flowers	4.93	0.085	0.010	0.240	6.22	0.045	0.015	0.239	2.93	0.231	0.011	0.014	2.05	0.359	0.011	0.185
Biomass					5.57	0.062	0.014	0.117					0.02	0.992	0.000	0.052
Response variable			Sympatric-Absent													
Survival	111.14	**<0.001**	0.146	0.274	3.72	0.054	0.008	0.081	44.12	**<0.001**	0.083	0.402	27.15	**<0.001**	0.040	0.114
Plant height	2.29	0.130	0.006	0.592	28.51	**<0.001**	0.122	0.332	2.06	0.151	0.012	0.364	5.53	0.019	0.069	0.132
Number of ramets	3.30	0.069	0.014	0.210	27.95	**<0.001**	0.121	0.324	2.80	0.094	0.023	0.042	4.74	0.030	0.055	0.060
Number of flowers	4.93	0.085	0.010	0.240	6.22	0.045	0.015	0.239	2.93	0.231	0.011	0.014	2.05	0.359	0.011	0.185
Biomass					47.74	**<0.001**	0.172	0.455					20.43	**<0.001**	0.228	0.319

**Table 2.** Results of mixed-effects models testing for local adaptation along an environmental difference gradient in *Anemone nemorosa* and *Milium effusum* in 2022. We used performance traits in *A. nemorosa* and *M. effusum* for 2022 at sympatric and allopatric sites as response variables, and the linear and quadratic terms of environmental differences (Δ) between the site of origin and the site of transplantation as fixed factors. As response variables we tested survival, plant height, number of ramets, number of flowers and biomass, and as environmental variables we included structural management intensity (SMI), structural complexity, soil pH and mean annual spring temperature, where the quadratic form is indicated with a superscripted 2 behind the variable name. Region, population and genet were set as nested random factors (residual variances are ranging from 0.013 to 208.78). Marginal *R*^2^ (*R*_m_) and conditional *R*^2^ (*R*_c_) values for the model are given. Significant *P* values after applied Bonferroni-correction are indicated in bold.

		*Anemone nemorosa*				*Milium effusum*			
		*χ* ^2^	*P* value	*R*_*c*_	*R*_*m*_	*χ* ^2^	*P* value	*R*_*c*_	*R*_*m*_
Response variable	Explanatory variable								
Survival	ΔSMI	38.75	**<0.001**	0.352	0.159	4.50	0.034	0.335	0.188
	ΔSMI^2^	0.35	0.553			10.29	**0.001**		
	Δstructural complexity	8.01	0.005			14.39	**<0.001**		
	Δstructural complexity^2^	0.01	0.911			1.52	0.218		
	Δsoil pH	42.09	**<0.001**			7.17	0.007		
	Δsoil pH^2^	0.76	0.384			4.88	0.027		
	Δspring temperature	11.53	**<0.001**			0.56	0.454		
	Δspring temperature^2^	3.18	0.074			0.25	0.620		
Plant height	ΔSMI	35.62	**<0.001**	0.543	0.267	1.06	0.304	0.667	0.284
	ΔSMI^2^	3.82	0.051			1.28	0.258		
	Δstructural complexity	9.15	**0.002**			0.01	0.936		
	Δstructural complexity^2^	6.45	0.011			1.04	0.309		
	Δsoil pH	0.53	0.469			41.86	**<0.001**		
	Δsoil pH^2^	0.00	0.997			4.08	0.044		
	Δspring temperature	40.92	**<0.001**			0.98	0.323		
	Δspring temperature^2^	3.06	0.080			0.82	0.365		
Number of ramets	ΔSMI	20.40	**<0.001**	0.310	0.188	0.43	0.512	0.354	0.098
	ΔSMI^2^	0.91	0.342			0.06	0.803		
	Δstructural complexity	0.13	0.722			0.22	0.641		
	Δstructural complexity^2^	8.74	**0.003**			4.12	0.042		
	Δsoil pH	19.21	**<0.001**			5.39	0.020		
	Δsoil pH^2^	0.00	0.981			0.29	0.593		
	Δspring temperature	15.19	**<0.001**			1.44	0.230		
	Δspring temperature^2^	1.93	0.165			0.16	0.691		
Number of flowers	ΔSMI	2.17	0.140	0.341	0.066	0.19	0.665	0.336	0.194
	ΔSMI^2^	5.52	0.019			0.32	0.575		
	Δstructural complexity	0.99	0.320			1.44	0.230		
	Δstructural complexity^2^	5.58	0.018			0.08	0.781		
	Δsoil pH	2.15	0.143			0.13	0.717		
	Δsoil pH^2^	2.24	0.135			5.36	0.021		
	Δspring temperature	0.11	0.738			0.97	0.325		
	Δspring temperature^2^	0.21	0.650			0.93	0.336		
Biomass	ΔSMI	48.26	**<0.001**	0.430	0.223	0.44	0.506	0.619	0.292
	ΔSMI^2^	4.64	0.031			0.40	0.529		
	Δstructural complexity	2.17	0.141			0.11	0.736		
	Δstructural complexity^2^	8.84	0.003			1.75	0.186		
	Δsoil pH	26.26	**<0.001**			43.23	**<0.001**		
	Δsoil pH^2^	0.33	0.565			1.34	0.248		
	Δspring temperature	10.11	**0.001**			4.38	0.036		
	Δspring temperature^2^	1.92	0.166			1.01	0.315		

**Table 3.** Results of mixed-effects models testing for recruitment vs. dispersal limitations in *Anemone nemorosa* and *Milium effusum* in 2022. We used performance traits in *A. nemorosa* and *M. effusum* for 2022 at sympatric and absent sites as response variables, and the linear and quadratic terms of environmental differences (Δ) between the site of origin and the site of transplantation as fixed factors. As response variables we tested survival, plant height, number of ramets, number of flowers and biomass, and as environmental variables we included structural management intensity (SMI), structural complexity, soil pH and mean annual spring temperature, where the quadratic form is indicated with a superscripted 2 behind the variable name. Region, population and genet were set as nested random factors (residual variances are ranging from 0.013 to 208.78). Marginal *R*^2^ (*R*_*m*_) and conditional *R*^2^ values for the model is given. Significant *P* values after applied Bonferroni-correction are indicated in bold.

Response variable	Explanatory variable	*Anemone nemorosa*				*Milium effusum*			
		*χ* ^2^	*P* value	*R*_*c*_	*R*_*m*_	*χ* ^2^	*P* value	*R*_*c*_	*R*_*m*_
Survival	ΔSMI	0.24	0.624	0.514	0.297	3.91	0.048	0.795	0.780
	ΔSMI^2^	8.97	0.003			7.50	0.006		
	Δstructural complexity	2.06	0.151			0.38	0.536		
	Δstructural complexity^2^	3.11	0.078			1.53	0.217		
	Δsoil pH	4.41	0.036			0.98	0.322		
	Δsoil pH^2^	0.05	0.819			1.54	0.215		
	Δspring temperature	0.25	0.617			2.16	0.141		
	Δspring temperature^2^	0.17	0.677			21.66	**<0.001**		
Plant height	ΔSMI	2.12	0.146	0.222	0.649	0.75	0.385	0.419	0.776
	ΔSMI^2^	1.93	0.164			3.50	0.061		
	Δstructural complexity	17.15	**<0.001**			2.21	0.137		
	Δstructural complexity^2^	1.31	0.253			1.28	0.258		
	Δsoil pH	0.56	0.452			3.00	0.083		
	Δsoil pH^2^	1.00	0.317			3.61	0.058		
	Δspring temperature	0.02	0.895			0.13	0.717		
	Δspring temperature^2^	0.03	0.871			2.36	0.124		
Number of ramets	ΔSMI	2.76	0.097	0.176	0.472	0.04	0.847	0.319	0.499
	ΔSMI^2^	1.43	0.232			0.04	0.838		
	Δstructural complexity	0.05	0.817			1.07	0.301		
	Δstructural complexity^2^	0.47	0.494			0.19	0.665		
	Δsoil pH	4.51	0.034			1.72	0.189		
	Δsoil pH^2^	0.09	0.768			2.65	0.103		
	Δspring temperature	0.17	0.681			0.41	0.524		
	Δspring temperature^2^	1.33	0.248			0.52	0.472		
Number of flowers	ΔSMI	0.08	0.778	0.999	0.999	0.10	0.756	0.624	0.394
	ΔSMI^2^	0.31	0.575			0.52	0.471		
	Δstructural complexity	0.00	0.954			0.84	0.358		
	Δstructural complexity^2^	0.00	0.975			0.02	0.897		
	Δsoil pH	0.01	0.942			1.17	0.279		
	Δsoil pH^2^	0.00	0.986			1.30	0.254		
	Δspring temperature	0.00	0.977			0.07	0.799		
	Δspring temperature^2^	0.00	0.984			1.31	0.252		
Biomass	ΔSMI	1.64	0.201	0.193	0.609	0.18	0.669	0.309	0.665
	ΔSMI^2^	0.39	0.530			0.77	0.381		
	Δstructural complexity	0.50	0.478			1.66	0.198		
	Δstructural complexity^2^	3.14	0.076			0.29	0.588		
	Δsoil pH	4.31	0.038			1.40	0.238		
	Δsoil pH^2^	0.02	0.891			2.03	0.155		
	Δspring temperature	3.04	0.081			0.03	0.859		
	Δspring temperature^2^	3.11	0.078			0.94	0.333		

Overall, survival decreased, whereas plant height, number of ramets and biomass generally increased when transplanted to sites that had lower management intensity and that were colder, more acidic and more structurally complex. Two quadratic relationships were found, one with Δstructural complexity in *A. nemorosa* and one with ΔSMI in *M. effusum*, both relationships were concave, indicating local adaptation.

### Sympatric-absent comparisons

**Figure 7.**
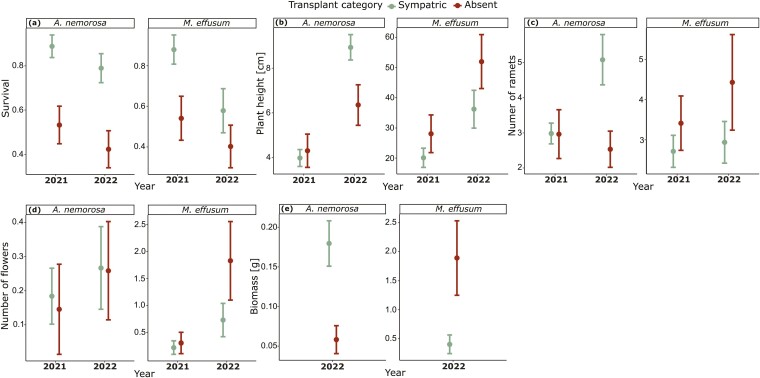
Plant traits in 2021 and 2022 for the sympatric and absent transplantation sites for *Anemone nemorosa* and *Milium effusum.* (A) survival, (B) plant height, (C) number of ramets, (D) number of flowers and (E) aboveground biomass. Circles indicate means across populations, and the error bars indicate the standard error.

Mortality differed significantly between sympatric and absent sites in both species in 2021 and for *M. effusum* in 2022, with the highest survival at sympatric sites (Fig. 2; Table 1). Tests for population differences between the sympatric and absent transplant sites revealed scarcely significant differences in the other measured traits from either species in 2021 [**see Supporting Information—Table S3**], whereas, in 2022, we found significant differences in plant height, number of ramets and biomass for *A. nemorosa* and in biomass for *M. effusum* (Table 1). In general, the observed significant effects were stronger in *A. nemorosa* than in *M. effusum* (Fig. 7). Moreover, the species generally showed opposing patterns in all measured traits except survival: *A. nemorosa* populations performed better in sympatric sites, whereas *M. effusum* populations performed better in absent sites; survival was higher in sympatric sites in both species (Fig. 7).

Testing for effects of environmental differences (Δ) between population origins and absent transplant sites on the five measured traits in 2022, we only found few statistically significant relationships. *Anemone nemorosa* was significantly affected in plant height by the linear term of Δstructural complexity (Table 3). Furthermore, *M. effusum* had a significant quadratic effect of Δspring temperature on survival (Table 3).

## Discussion

We reveal some evidence of local adaptation via the sympatric–allopatric comparisons in *A. nemorosa* and *M. effusum* to forest structural attributes and the forest microenvironment. However, most relationships indicate plastic responses of performance traits to local environmental conditions, revealing the importance of phenotypic plasticity in shaping plant phenotypes along environmental gradients. Considering the sympatric-absent comparisons, in contrast to *A. nemorosa* performing best at their sympatric sites, *M. effusum* performed best at previously unoccupied sites, indicating recruitment limitation in the former and dispersal limitation in the latter species.

### Sympatric–allopatric comparisons

Differences in mortality between sympatric, near allopatric and far allopatric sites are small in both years for both species compared to differences between sympatric and absent sites. For *A. nemorosa* a small portion of plants died in both 2021 and 2022, whereas for *M. effusum* the percentage of mortality was higher, especially in 2022. In our design, the two species shared most of the origin and transplant sites and thus experienced the same environmental conditions in both years, excluding different environments between species as an explanation for the observed differences in mortality. However, the species differ in their life history, which may explain mortality discrepancies. *Anemone nemorosa* is a spring geophyte and thus has dormant rhizomes belowground for most of the year, well protected from potential droughts. In contrast, *M. effusum* is exposed to the aboveground environment all year round, rendering it more vulnerable to, for example, summer droughts or frost damage, which may explain the higher mortality. In fact, soil moisture was lower in 2022 than in 2021 in the critical summer months just before measurements of *M. effusum* were taken [**see Supporting Information—Fig. S4**].

Aside from mortality, sympatric–allopatric comparisons yielded hardly any differences for any of the other measured traits, potentially due to the implementation of artificial categories of sympatric and (near and far) allopatric sites based on the SMI gradient. Silvicultural management intensity is a complex index derived from a mix of factors (tree species, stand age, above and below-ground dead wooden biomass) that, each on their own, may have their own effects on abiotic environmental variables and thus on selection pressures on the plants. Indeed, when we switch to a continuous approach and use environmental differences between origin and transplant sites for several environmental variables, many linear and some quadratic relationships between our measured traits and the environmental difference between home and transplant site became apparent.

Only a few sympatric–allopatric relationships along the continuous gradient were present in 2021, while many significant relationships were found in 2022. This is likely because the individuals were establishing in their transplanted environment in 2021 and time was still too short to accrue phenotypic differences that reflect the transplant environment. Other studies likewise show that several years can be necessary in order to detect local adaptation (Bennington *et al.* 2012; Johnson *et al.* 2022).

In 2022, the significant relationships between the measured traits and the forest structural attributes show the ability of the studied understorey herbs to respond to environmental features. Most of these relationships were linear, indicating that fitness is not highest at the sympatric sites but rather that plastic responses shape phenotypic expression along environmental gradients in a linear way (Gorton *et al*. 2022). Ultimately, this indicates that more favourable environmental conditions can cause improved performance along an environmental gradient. All measured traits in *A. nemorosa* increased when transplanted to sites with lower SMI, soil pH and spring temperature. Lower forest management intensity can be tightly linked to higher structural complexity and might promote increased trait performance due to increased environmental heterogeneity in the forest stand and more light influx. The observed phenotypic plasticity may thus be an indirect response to forest management via the microclimate (Willems *et al.* 2021; Møller *et al.* 2023a). Similarly, lower pH also benefitted plant height and biomass in *M. effusum*. Lower soil pH correlates with colder mean annual spring temperature (Møller *et al.* 2023a), with both possibly being driven by the proportion of conifers in the forest stand (Willems *et al.* 2021), and in the face of global warming, the optimal thermal niche in the studied regions might be increasingly more located in coniferous sites, which are relatively cold. Previous studies have found similar evidence of adaptive shifts attributed to recent climatic changes (Wilczek *et al.* 2014; Anderson and Wadgymar 2020). Furthermore, the concave quadratic effects of structural complexity observed in number of ramets in *A. nemorosa* in 2022 suggest that this specific forest structural attribute (or any environmental factor correlating with it, such as light regime) is an important driver of local adaptation in forest understorey herbs.

The detection of some local adaptation at the scale of the three investigated regions indicates the evolutionary potential to adapt to a geographic mosaic of structural complexity related to forest management intensity and may suggest that specifically *A. nemorosa* has been subject to strong selective forces. Furthermore, the absence of effects on our only sexual reproductive trait, number of flowers, indicates that phenotypic plasticity is primarily acting on vegetative rather than on flowering traits. A reason for this could be that the number of flowers was generally still low, and it may take more years until differential patterns between transplant types or along environmental gradients would appear in this trait (Bennington *et al.* 2012). Similar transplant experiments where local populations have been transplanted along an environmental gradient have produced a variety of outcomes. Often the local genotype performs best in its sympatric site, indicating strong local adaptation (Ågren and Schemske 2012; Bennington *et al.* 2012; De Frenne *et al.* 2012; Anderson *et al.* 2013; Toräng *et al.* 2015). However, phenotypic plasticity resulting in increasingly altered trait values under increasingly changed environmental conditions along transplant sites are also common (Pluess *et al.* 2011; Meineri *et al.* 2013). Our observation of phenotypic plasticity displayed via significant linear relationships between the majority of traits and environmental differences between the transplant sites and the sites of origin may on the one hand reflect passive responses to ecologically important environmental factors. On the other hand, it may also reflect the plants’ opportunistic responses to benign conditions, helping to adjust to a diverse range of environmental conditions and act beneficial for local adaptation as a precursor (Radersma *et al.* 2020).

### Sympatric-absent comparisons

The mortality occurring in 2021 mainly occurred on the absent sites, where both species did not naturally occur, which indicates that these absent sites are primarily unsuitable conditions for the study species. This pattern was also visible in most traits for *A. nemorosa* and some in *M. effusum* in the categorical sympatric-absent models. However, the scarcity of significant relationships between the measured performance traits and the environmental differences in the sympatric-absent models indicates that we can rule out that the specific environmental variables included in this study caused the sites to be unsuitable. Since the fundamental niche of a species is generally broader than the realized niche, further research could consider the role of biotic factors such as herbivory, plant–soil biota interactions or pathogens as explanations for the species’ absence on these sites (Nooten and Hughes 2017).

When comparing the measured performance traits between the categories sympatric and absent in 2021, no significant differences were observed for either species, but these effects were striking in 2022 (Table 1, Fig. 7). In 2022, whereas *A. nemorosa* individuals performed better at sympatric sites, *M. effusum* showed the exact opposite pattern, performing better when transplanted to sites where they did not naturally occur. One potential explanation of this increased fitness in absent sites can be the result of a release from local enemies, such as soil pathogens and below-ground herbivores (De Frenne *et al.* 2014a). As an alternative explanation, *M. effusum* individuals only performed better at absent sites in 2022 after a big mortality event in 2021, which could have acted as a strong selection event. If only the largest, most fit individuals survived and established under the supposedly harsher environmental conditions, these could subsequently outperform, on average, individuals transplanted to more benign sympatric sites where genotypes with lower performance still managed to survive. Taken together, these results hint towards *A. nemorosa* being mostly recruitment limited as evidenced by consistently poorer performance at absent sites (Ehrlén and Eriksson 2000), whereas *M. effusum*, once established, performed better at absent sites which suggests dispersal limitation.

### Both species show minor local adaptation to forest management on a regional scale

The environmental variables investigated in this study are either tightly linked to and affected by forest management or they are themselves reflecting management intensity (Willems *et al.* 2021; Møller *et al.* 2023a). Based on the significant concave relationship of number of ramets with structural complexity in *A. nemorosa* and survival with SMI in *M. effusum*, we have two cases of forest understorey herbs showing local adaptation to their sympatric forest patch embedded within a geographic mosaic of environments. Structural complexity is a representation of vertical and horizontal structural heterogeneity, which is often lacking in forests with high management intensity. A heterogeneous microenvironment can select for phenotypic variation within plant traits (Møller *et al.* 2023a) and since structural complexity reflects strong spatial and temporal environmental variation, populations could be expected to adapt to that. On the other side of the spectrum, low structural complexity, as is typical in homogeneous spruce forest environments, may demand its own specialized adaptations to this monotonous environment.

Land use has been recognized as having one of the strongest impact on species and genetic diversity (IPCC 2014). It is critical to test how individuals originating from various management intensities can potentially establish in nonlocal sites for metapopulations to persist in our rapidly changing world. In contrast to previous studies operating at larger scales (De Frenne *et al.* 2012; Anderson *et al.* 2013; Toräng *et al.* 2015), our study takes place on a regional scale, containing a heterogeneous mosaic of forest management intensities and types, and we revealed genetically based phenotypic variation at this scale (see also Møller *et al.* 2023a) as well as some evidence for local adaptation at the regional level. However, it is important to keep in mind that despite our attempts to keep rhizome fragments and ramet sizes consistent across origin and transplant treatments, part of the observed variation might still be attributed to epigenetics or maternal effects (which should have dwindled over time (Ouborg *et al.* 1991; Schmid and Dolt 1994)). Furthermore, the sympatric-absent comparisons give insights on the dispersal vs. recruitment limitations of the two species in temperate forests and thus provide useful discernments for their responses to environmental changes as well as valuable information within the framework of ecological restoration practices.

## Conclusions

In sum, our experiment shows that variation in forest structural complexity and SMI (silvicultural management intensity) can lead to local adaptation. However, more prominent were our observations that variation in forest management and the forest microenvironment caused substantial linear phenotypic plastic responses in performance traits, highlighting that the forest understorey environment strongly affects plant fitness. Possibly, the observed phenotypic plasticity may be an indirect response to forest management affecting the microclimate. In addition, we found that the two studied forest understorey herbs varied in their strength of phenotypic plasticity and also in their ability to establish in unoccupied sites. Ultimately, our results contribute to the field of forest management and ecological restoration by shedding light on the evolutionary and ecological forces driving intraspecific variation in understorey herbs.

## Supporting Information

The following additional information is available in the online version of this article –

**Table S1.** List of the selected plots, ID, region, forest management intensity (SMI) and the allocated SMI bin.

**Table S2.** Near allopatric and far allopatric category comparisons for traits measured in 2022.

**Table S3.** Results of mixed-effects models of the measured traits in Anemone nemorosa and Milium effusum for 2021 for sympatric versus allopatric and sympatric versus absent comparisons.

**Figure S1.** Photos of the pots in the reciprocal transplant experiment in the field.

**Figure S2.** Correlation matrix for the measured plant traits.

**Figure S3.** Correlation matrix for the environmental variables.

**Figure S4.** Soil moisture count at plots where mortality of *Milium effusum* was high.

**R code.** R code for replicating all statistical analyses.

plae061_suppl_Supplementary_Tables_S1-S3_Figures_S1-S4

plae061_suppl_Supplementary_Materials

## Data Availability

This work is based on data elaborated by the HerbAdapt and Forest Structure (core) projects of the Biodiversity Exploratories program (DFG Priority Program 1374). The datasets are publicly available in the Biodiversity Exploratories Information System (http://doi.org/10.17616/R32P9Q, https://www.bexis.uni-jena.de/ddm/data/Showdata/22766 and https://www.bexis.uni-jena.de/ddm/data/Showdata/31469).

## Statement of Inclusion

Our study brings together authors from different countries, including scientist based in the country where the study was carried out. If no engagement in the early stages of the research and study design was possible, authors were engaged in the data analyses and manuscript writing stages to ensure that all authors felt heard and diverse perspectives were considered. Whenever relevant, literate published by scientists from the region was cited; efforts were made to consider relevant work published in the local language.
