# Supplementary material for: Local adaptation and phenotypic plasticity in two forest understorey herbs in response to forest management intensity
Source: AoB Plants. 2024 Nov 21;17(1):plae061. doi: 10.1093/aobpla/plae061 (PMC11752646; doi:10.1093/aobpla/plae061)
Supplement: plae061_suppl_Supplementary_Tables_S1-S3_Figures_S1-S4 [file plae061_suppl_supplementary_tables_s1-s3_figures_s1-s4.pdf]

Supporting Information

Table S1. Selected plots from the three regions: Schwäbische Alb (ALB), Hainich-Dün (HAI), and Schorfheide-Chorin (SCH) with their plot numbers within the Biodiversity Exploratories, the corresponding forest management intensity (SMI) as well as the assigned SMI bin. Letters behind bins indicate if the plots were used only for *Anemone nemorosa* (A), only for *Milium effusum* (M), or for both (nothing).

Plot ID	Region	SMI	SMI bin
AEW8	ALB	0.011	Low (A)
AEW12	ALB	0.602	High
AEW15	ALB	0.406	Absent (A)
AEW21	ALB	0.124	Low
AEW25	ALB	0.418	Absent (M)
AEW29	ALB	0.487	High
AEW31	ALB	0.538	Absent (A)
AEW33	ALB	0.528	High
AEW36	ALB	0.421	Mid
AEW37	ALB	0.301	Mid
AEW40	ALB	0.119	Low (M)
AEW41	ALB	0.172	Low
AEW42	ALB	0.241	Mid
AEW44	ALB	0.292	Absent (M)
AEW50	ALB	0.134	Absent
HEW3	HAI	0.508	Absent
HEW9	HAI	0.188	Mid
HEW13	HAI	0.389	Absent
HEW16	HAI	0.333	Mid
HEW17	HAI	0.364	High
HEW19	HAI	0.071	Low
HEW21	HAI	0.135	Low (A)
HEW23	HAI	0.397	High
HEW28	HAI	0.270	Mid (A)
HEW33	HAI	0.047	Low (A)
HEW36	HAI	0.130	Low (M)
HEW44	HAI	0.486	High
SEW6	SCH	0.319	High (A)

SEW7	SCH	0.082	Low (A)
SEW8	SCH	0.059	Low (A)
SEW23	SCH	0.162	Mid (A)
SEW26	SCH	0.051	Absent (A)
SEW35	SCH	0.234	Absent (A)
SEW36	SCH	0.166	Absent (A)
SEW40	SCH	0.250	High (A)
SEW43	SCH	0.229	High (A)
SEW46	SCH	0.088	Mid (A)
SEW47	SCH	0.082	Low (A)
SEW50	SCH	0.116	Mid (A)

7

Table S2. Near allopatric and far allopatric category comparisons for traits measured in 2022. The measured traits are set as the response variables, and category with two levels (near and far) is set as the explanatory factor. No P-values were significant after Bonferroni correction.

		Anemone nemorosa		*Milium effusum*	
**Response variable**	**Explanatory variable**	χ^2	**P-value**	χ^2	**P-value**
Survival	Transplant category (near – far)	3.721	0.054	0.001	0.977
Plant height		4.669	0.031	0.234	0.629
Number of ramets		1.585	0.208	0.032	0.856
Flower score		7.546	0.006	1.655	0.198
Biomass		6.244	0.012	0.216	0.642

Table S3. Results of mixed-effects models of the measured traits in *A. nemorosa* and *M. effusum* for 2021 at 'Sympatric & Allopatric' and 'Sympatric & Absent' transplantation sites as response variables, and the linear and quadratic terms of environmental and microclimatic variables as fixed factors. Population, genet, and region were set as random factors. Significant P-values after Bonferroni correction are indicated in bold.

		Anemone nemorosa				*Milium effusum*			
		**Sympatric-Allopatric**		**Sympatric-Absent**		**Sympatric-Allopatric**		**Sympatric-Absent**	
**Response variable**	**Explanatory variable**	χ^2	P-value	χ^2	P-value	χ^2	P-value	χ^2	P-value
Survival	SMI	0.674	0.412	0.854	0.355	2.871	0.090	3.237	0.072
	SMI^2	3.411	0.065	1.833	0.176	1.308	0.253	5.690	0.017
	Structural complexity	0.974	0.323	1.908	0.167	0.643	0.423	0.347	0.557
	Structural complexity^2	2.629	0.105	1.070	0.301	0.173	0.678	4.608	0.032
	Soil pH	0.073	0.787	1.553	0.213	1.597	0.206	0.103	0.749
	Soil pH^2	1.173	0.279	0.758	0.384	0.144	0.704	1.667	0.197
	Spring temperature	3.624	0.057	0.189	0.664	1.248	0.264	0.675	0.411
	Spring temperature^2	1.006	0.316	2.916	0.088	0.027	0.870	5.698	0.017
Plant height	SMI	1.341	0.247	1.787	0.181	5.033	0.025	0.046	0.830
	SMI^2	1.386	0.239	1.247	0.264	0.197	0.657	1.082	0.298
	Structural complexity	1.650	0.199	17.13	**<0.001 *****	6.011	0.014	1.340	0.247
	Structural complexity^2	0.644	0.422	0.741	0.390	0.076	0.782	1.573	0.210
	Soil pH	0.003	0.960	0.454	0.500	15.02	**<0.001 *****	3.625	0.057
	Soil pH^2	0.106	0.744	1.084	0.298	5.103	0.024	4.060	0.044
	Spring temperature	4.070	0.044	0.022	0.882	7.595	**0.006 ***	0.006	0.937
	Spring temperature^2	0.991	0.319	0.017	0.897	0.002	0.964	1.068	0.301
Number of ramets	SMI	6.648	0.010	2.751	0.097	4.302	0.038 *	0.126	0.723
	SMI^2	0.427	0.513	1.349	0.245	0.027	0.870	0.001	0.973
	Structural complexity	0.046	0.830	0.058	0.810	1.757	0.185	0.402	0.526
	Structural complexity^2	0.019	0.891	0.439	0.508	2.048	0.152	0.422	0.516
	Soil pH	0.982	0.322	4.470	0.034	1.209	0.271	1.902	0.168
	Soil pH^2	1.960	0.161	0.101	0.750	0.116	0.733	2.757	0.097
	Spring temperature	0.499	0.480	0.180	0.671	2.001	0.157	0.165	0.684
	Spring temperature^2	0.009	0.924	1.290	0.356	0.019	0.891	0.200	0.655
Number of flowers	SMI	0.412	0.521	0.080	0.778	0.013	0.908	0.000	0.984
	SMI^2	3.674	0.055	0.314	0.575	1.369	0.242	0.004	0.949
	Structural complexity	2.250	0.134	0.003	0.954	1.955	0.162	1.536	0.215
	Structural complexity^2	0.011	0.919	0.001	0.975	0.351	0.553	1.351	0.245

	Soil pH	10.437	**0.001 ****	0.005	0.942	3.474	0.062	0.266	0.606
	Soil pH^2	0.288	0.592	0.000	0.986	0.328	0.567	0.145	0.704
	Spring temperature	5.504	0.019	0.001	0.977	1.809	0.179	1.195	0.274
	Spring $temperature^2$	4.512	0.034	0.000	0.984	2.210	0.137	0.375	0.540

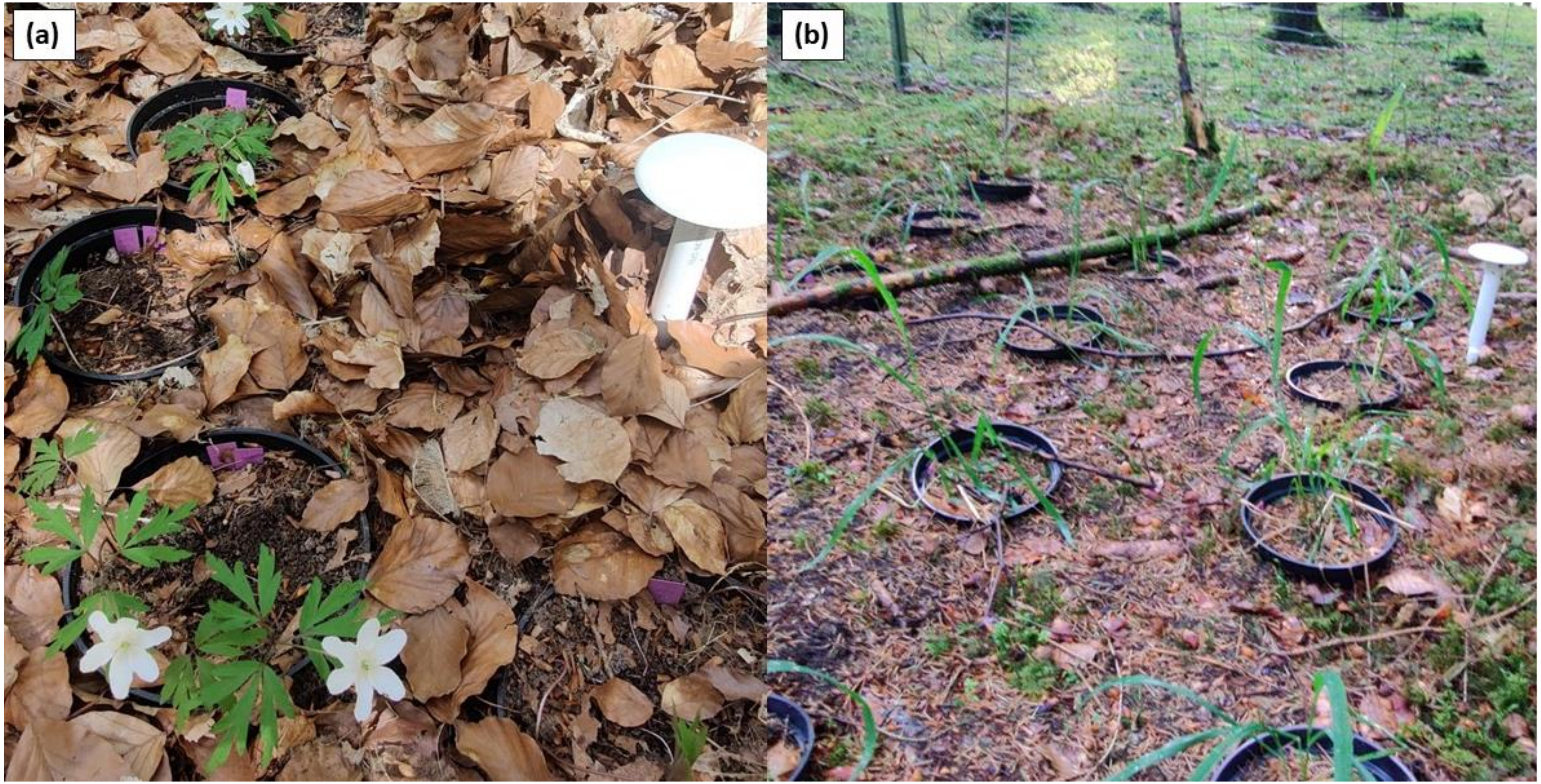

Figure S1. Photos of the pots in the reciprocal transplant experiment in the field, demonstrating how pots were dug in at ground level of **(a)** *Anemone nemorosa* and **(b)** *Milium effusum*.

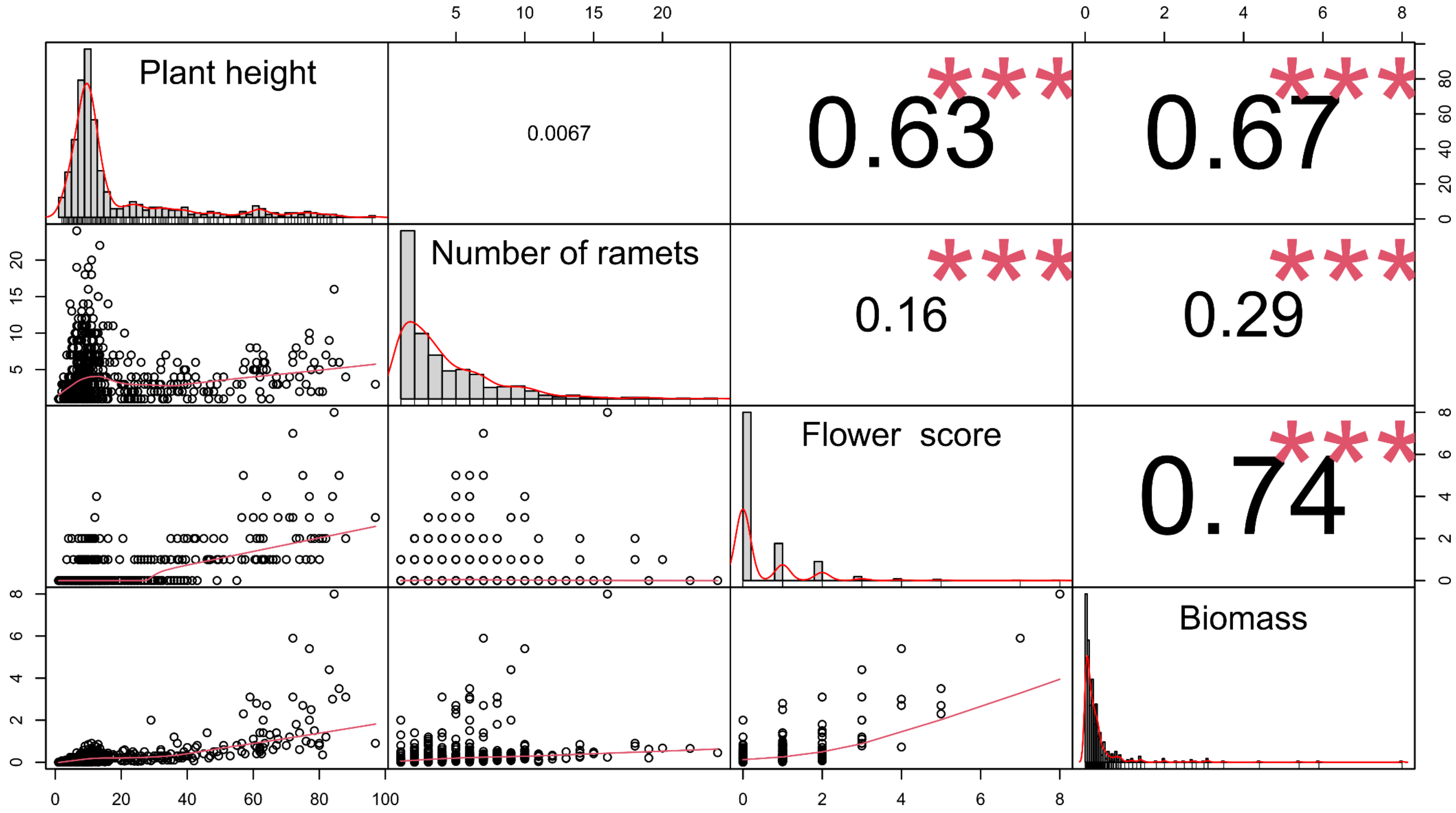

Figure S2. Correlation matrix for the measured plant traits: plant height, number of ramets, flower score, and biomass. Diagonal squares show the distribution of each variable in the form of histograms for the individual traits. Lower triangular squares show scatter plots between the pairwise relationships. The upper triangular squares show the correlation coefficients (r-values) for each pair of variables. Statistical significance is indicated by asterisks.

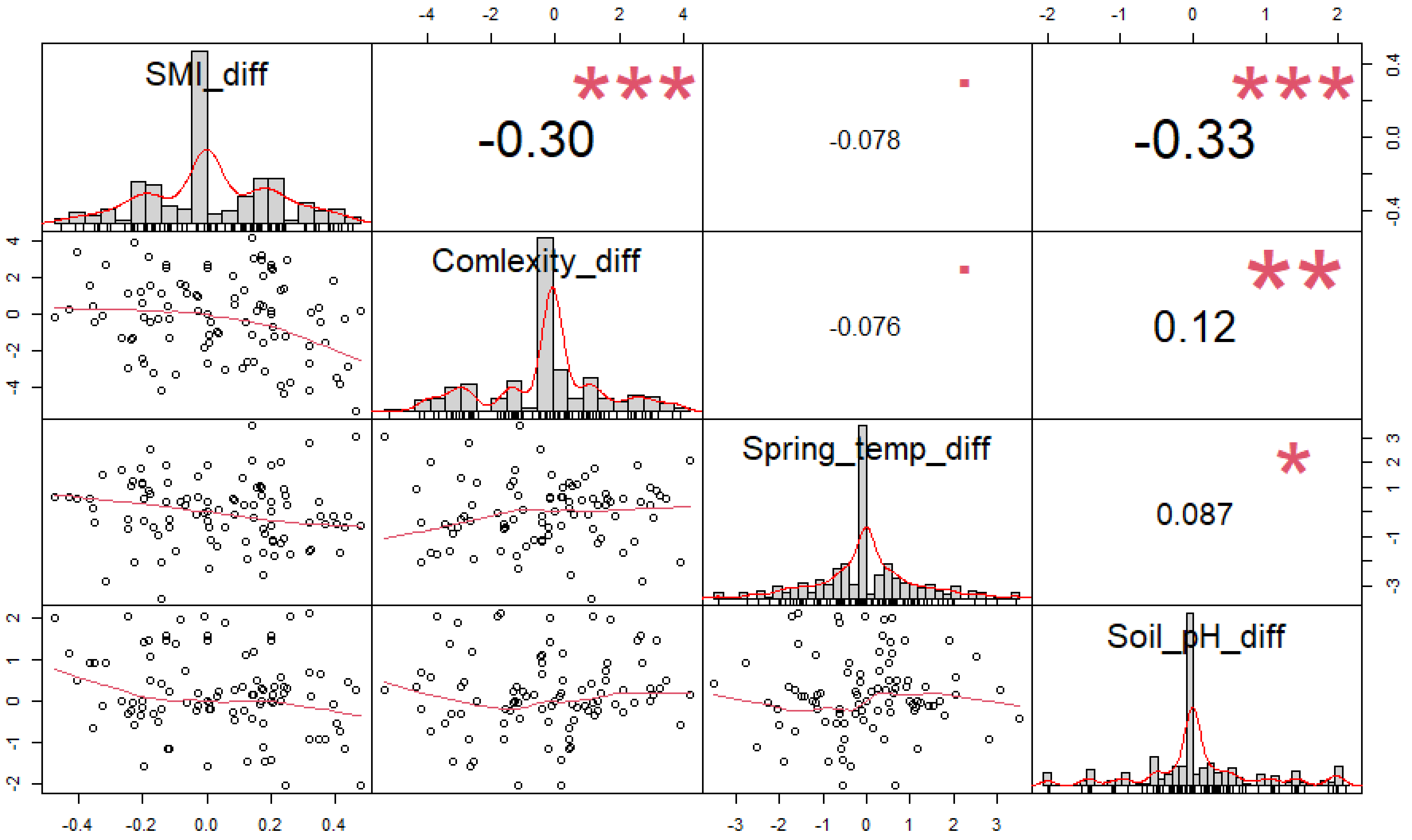

Figure S3. Correlation matrix for the environmental variables: SMI_diff (ΔSMI), Complexity_diff (ΔStructural complexity), Spring_temp_diff (ΔSpring temperature), and Soil_pH_diff (ΔSoil pH). Diagonal squares show the distribution of each variable in the form of histograms for the environmental variables. Lower triangular squares show scatter plots between the pairwise relationships. The upper triangular squares show the correlation coefficients (r-values) for each pair of variables. Statistical significance is indicated by asterisks.

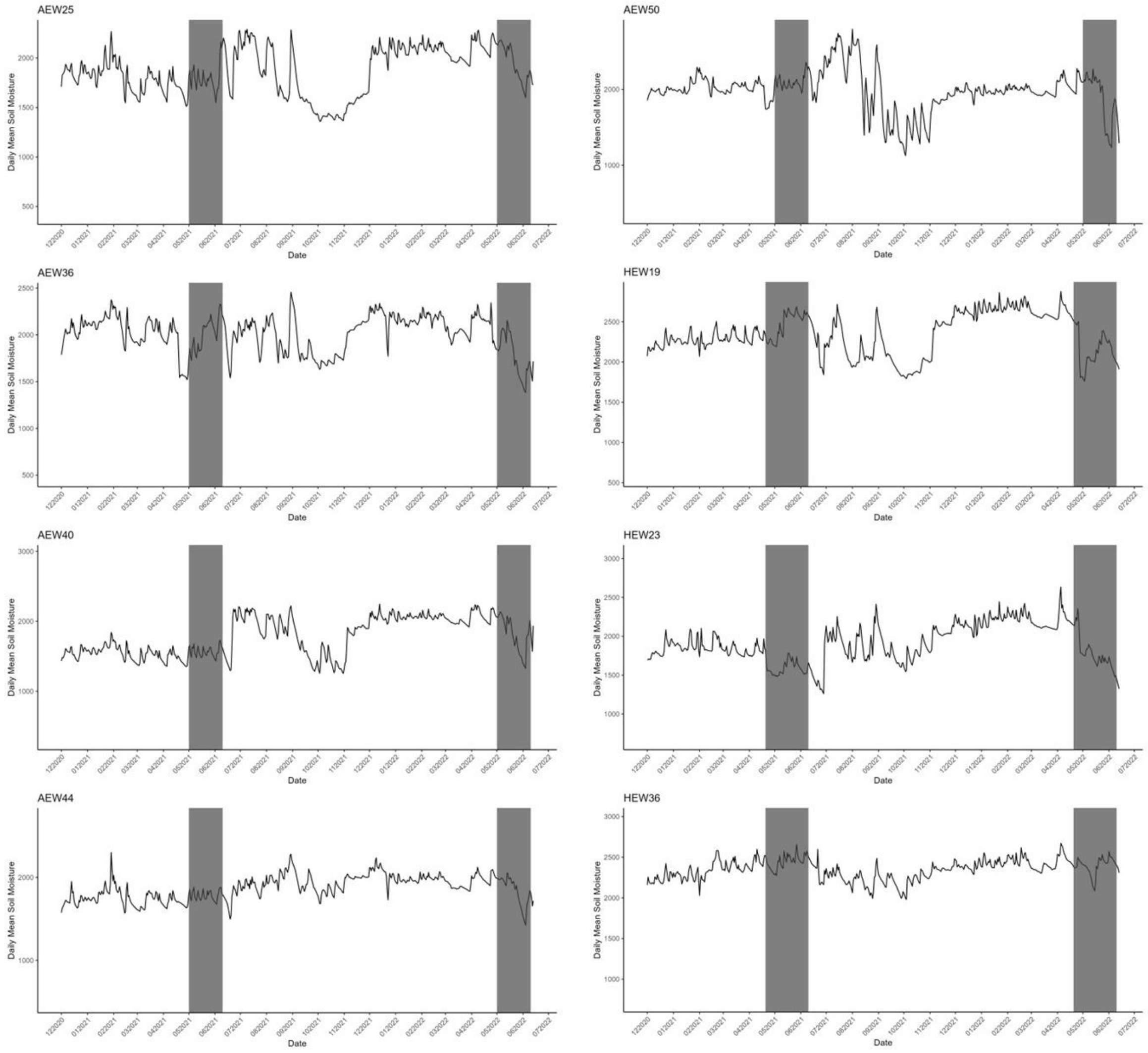

Figure S4. Soil moisture count from December 2020 to June 2022, at four plots in the Schwäbische Alb (AEW25, AEW36, AEW40, AEW44 and AEW50) and one plot in Hanich-Dün (HEW19, HEW23, and HEW36) where mortality of *Milium effusum* was high between measurements taken in 2021 and 2022. Data extracted from TOMST TMS4 loggers. Grey highlighted area compare the month of May in 2021 and 2022, indicating a potential drought in 2022.
